# Less Empathic and More Reactive: The Different Impact of Childhood Maltreatment on Facial Mimicry and Vagal Regulation

**DOI:** 10.1371/journal.pone.0163853

**Published:** 2016-09-29

**Authors:** Martina Ardizzi, Maria Alessandra Umiltà, Valentina Evangelista, Alessandra Di Liscia, Roberto Ravera, Vittorio Gallese

**Affiliations:** 1 Department of Neuroscience, University of Parma, Parma, Italy; 2 Ravera Children Rehabilitation Centre (RCRC), Lakka, Freetown, Sierra Leone; 3 Department of Pharmacy, University of Parma, Parma, Italy; 4 Department of Health Psychology, Hospital of Sanremo, Sanremo, Italy; 5 Institute of Philosophy, School of Advanced Study, University of London, London, United Kingdom; Harvard Medical School, UNITED STATES

## Abstract

Facial mimicry and vagal regulation represent two crucial physiological responses to others’ facial expressions of emotions. Facial mimicry, defined as the automatic, rapid and congruent electromyographic activation to others’ facial expressions, is implicated in empathy, emotional reciprocity and emotions recognition. Vagal regulation, quantified by the computation of Respiratory Sinus Arrhythmia (RSA), exemplifies the autonomic adaptation to contingent social cues. Although it has been demonstrated that childhood maltreatment induces alterations in the processing of the facial expression of emotions, both at an explicit and implicit level, the effects of maltreatment on children’s facial mimicry and vagal regulation in response to facial expressions of emotions remain unknown. The purpose of the present study was to fill this gap, involving 24 street-children (maltreated group) and 20 age-matched controls (control group). We recorded their spontaneous facial electromyographic activations of corrugator and zygomaticus muscles and RSA responses during the visualization of the facial expressions of anger, fear, joy and sadness. Results demonstrated a different impact of childhood maltreatment on facial mimicry and vagal regulation. Maltreated children did not show the typical positive-negative modulation of corrugator mimicry. Furthermore, when only negative facial expressions were considered, maltreated children demonstrated lower corrugator mimicry than controls. With respect to vagal regulation, whereas maltreated children manifested the expected and functional inverse correlation between RSA value at rest and RSA response to angry facial expressions, controls did not. These results describe an early and divergent functional adaptation to hostile environment of the two investigated physiological mechanisms. On the one side, maltreatment leads to the suppression of the spontaneous facial mimicry normally concurring to empathic understanding of others’ emotions. On the other side, maltreatment forces the precocious development of the functional synchronization between vagal regulation and threatening social cues facilitating the recruitment of fight-or-flight defensive behavioral strategies.

## Introduction

Childhood maltreatment, defined as “any act of omission or commission that results in harm or the potential for harm, regardless of intent” [1], represents a substantial threat to victims’ social skills development. The exposure to maltreatment during childhood causes brief- and long-term alterations of victims’ sense of self, emotion regulation, impulse control, social functioning and social relationships [2–4]. For example, strong associations between childhood abuse and externalizing problems, such as aggression and conduct disorders [5], as well as a tendency among abused children to show low rates of prosocial behaviors [6], have been extensively established.

These clinical observations are supported by numerous studies demonstrating, both at an explicit and implicit level, specific alterations in social cues processing, like facial expressions of emotions, among maltreated children and adolescents.

Underage victims of maltreatment preferentially recognize negative facial expressions of emotions, like fear and sadness, as anger [7,8]. Furthermore, angry facial expressions are also identified on the basis of less sensory inputs [9,10] and thanks to fewer expressive cues by physically abused children than controls [11].

Besides the explicit recognition of facial expressions, facial mimicry and autonomic regulation have specific relevance in the study of the processing of the facial expressions of emotions. On the one hand, facial mimicry represents the automatic, rapid and congruent electromyographic muscular reaction to others’ facial expressions [12,13] implicated in empathy, emotional reciprocity and emotions recognition [14–16]. Generally speaking, corrugator mimicry is facilitated during the observation of facial expressions of negative emotions and suppressed by positive ones [17]. Conversely, zygomaticus mimicry is promoted by facial expressions of positive emotions, like joy, and suppressed by negative ones. This positive-negative modulation is completely automatic [17] and related to valence attribution to others’ facial expressions of emotions [18,19]. Even if the majority of the studies about facial mimicry responses involved adult participants, the feasibility of the recording of facial mimicry responses to positive and negative facial expressions in children has been demonstrated [20–25].

On the other hand, autonomic regulation exemplifies the physiological adjustment to contingent environmental conditions (e.g., threatening or safe environments). Considering the autonomic adaptation to social contexts and social cues, vagal regulation performed by the myelinated branch of the vagus nerve is especially relevant. Indeed, the myelinated vagal pathway, unique in social mammals and originated in humans in the nucleus ambiguous, promotes the rapid inhibition of the sympathetic nervous system and dampens the hypothalamic-pituitary adrenal axis activity [26]. Furthermore, thanks to interneuronal connections, this myelinated branch of the vagus nerve also influences visceromotor efferent pathways necessary to express social engagement behaviors like looking (i.e., eyelid opening), emotional expressions (i.e., facial muscles), human voice extraction (i.e., middle ear muscles), verbal prosody (i.e., laryngeal and pharyngeal muscles), and social gestures (i.e., head turning muscles) [27]. Functionally, thanks to the modulation of both visceral and somatomotor states, the myelinated vagal pathway enables the rapid engagement and disengagement with objects and other people promoting a calm physiological state fostering social disposition [28]. The activity of this specific myelinated branch of the vagus nerve can be effectively quantified by the computation of Respiratory Sinus Arrhythmia (RSA; [29]) which is the periodic component of heart rate variability associated to respiratory frequency bands.

In spite of the importance of facial mimicry and vagal regulation in the study of the processing of the facial expression of emotions, few empirical studies investigated the possible alterations of these two physiological mechanisms after childhood maltreatment. Studies concerning facial mimicry reactions to others’ facial expression of emotions among maltreated children and adolescents obtained different results. Shackman and colleagues showed that physically abused children involved in a peer-directed aggression task exhibited greater corrugator mimicry and more aggressive behaviors when compared to age-matched controls [30]. Otherwise, a consistent suppression of corrugator mimicry in response to negative facial expressions of emotions was evidenced among abused street-boys when compared to age-matched controls [7] and among women victims of childhood sexual abuse [31].

Studies focused on maltreated children’s vagal regulation in response to facial expressions of emotions are still lacking. This issue was addressed only among maltreated adolescents, where the functional synchronization between vagal regulation and social external stimuli was assessed [7]. Higher baseline and greater RSA suppression are considered indexes of the functional ability to engage and disengage with the environmental requests [32]. Accordingly, people with higher baseline RSA should show greater RSA suppression to meet metabolic demands of taxing environmental conditions, including threatening social stimuli. The vagal suppression to hostile facial expressions (e.g., angry facial expression) therefore represents an adaptive and coherent autonomic modulation to external environmental stimuli which allows the functional recruitment of defensive behavioral strategies (e.g., fight/flight or immobilization). In a previous study on maltreated adolescents a dysfunctional vagal suppression in response to non-adverse facial expression of emotions was demonstrated [7]. Ineffective vagal regulation in children exposed to abuses was instead investigated not in response to facial expressions of emotions but in different social contexts revealing, also in this case, an abnormal vagal regulation. During the “strange situation task” neglected children showed an atypical increased RSA on separation and a decreased RSA on reunion [33]. Similarly, low RSA values during a joint task predicted the lowest inhibitory control among maltreated children [34]. These results suggest that children victims of maltreatment recruit ineffective defensive sympathetic behavioral strategies (e.g., fight/flight) when harmless social relations occur, but their vagal modulation in response to others’ facial expressions of emotions remains unknown.

From this concise review of the literature, significant alterations of the physiological substrates of empathic understanding of others’ emotions and of the self-regulation abilities in underage victims of maltreatment clearly appeared. However, the extremely limited and contradictory results concerning the effects of childhood maltreatment on facial mimicry and vagal regulation in response to facial expression of emotions among children demand further detailed studies.

The present study aims to fill this gap by investigating for the first time facial mimicry and vagal regulation to facial expressions of positive and negative emotions among street-children exposed to high levels of maltreatment. To this purpose, spontaneous facial electromyographic muscular activations (facial EMG) and RSA values of maltreated children and age-matched controls were recorded during the visualization of the facial expressions of anger, fear, joy and sadness. On the basis of the existing literature, differences between the two samples in corrugator muscular activations in response to negative facial expressions was expected. Furthermore, a dysfunctional negative correlation between RSA values at rest and RSA suppression values during the observation of non-adverse facial expressions of emotions was also anticipated among maltreated children but not among controls.

## Materials and Methods

### Participants

Sixty-four Sierra Leonean children were recruited for the study. Thirty-three were street-children collected directly from the street or in school enrolling abandoned children (Mal, maltreated group); 31 were control children engaged in private schools (Con, control group). The sample size exceeded the minimum amount required (n.36) estimated by means of statistical power analysis (a priori sample size n. evaluated for 1-ß = 0.95, α = 0.05 and effect size = 0.25). The sampling was suspended when two sex-balanced groups of enough size were obtained. All participants took also part in a previous behavioral study [8] conducted in a separate session. The general purposes and procedures of the study were orally explained by local social-workers to volunteers, and their legal guardians. If they agreed to participate to the study a written informed consent was collected. Participation in the study was completely voluntary, no participant has been repaid. All children filled a questionnaire through which their demographic information (i.e., sex, age, schooling, main language, ethnicity), actual and past life and health conditions (i.e., housing detail, necessities goods, history of tropical and infective pathologies, medical treatment), their socio-economic status (i.e., individual or family members’ income, occupation and education) and critical life events (i.e., sexual violence, physical violence, abuse, neglect, maltreatment, mourning) were obtained. Partial or unclear information was completed and checked thanks to sanitary, educational or charitable institutions. In order to control for between-groups differences in participants’ cognitive performance and naming skills, Colored Progressive Matrices (CPM) [35] and Boston Naming Test (BNT) [36] were administered. Participants who suffered from cardio-respiratory diseases and those who used drugs interfering with the cardio-respiratory activity were excluded from the sampling. Moreover, children who had more than 30% of trials rejected for artifacts were removed from the analyses (for further details about artifacts exclusion procedure please see below in “Procedure—facial EMG” paragraph). Two additional participants were subsequently removed due to their baseline RSA values being outliers (2 SD). The resulting final sample counted 44 participants. Of these 24 were maltreated children (Mal: mean age 7.71 years SE 0.32; mean years of schooling 2.50, SE 0.27; 12 males) and 20 were controls (Con: mean age 7.35 years SE 0.38; mean years of schooling 2, SE 0.23; 9 males). Maltreated children were all homeless street-children, they lived without a responsible adult and the street was their only source of basic needs (e.g., food, water, clothes, shelter). Control children were never been abandoned by their families, they lived with their parents or close relatives and they regularly attended to school. Among maltreated children, 62.5% experienced physical violence and 12.5% fell victim of sexual violence, finally 12.5% had suffered both physical and sexual violences. Otherwise, 10% of children belonging to Con group were exposed to physical violence and nobody experienced sexual violence. No significant difference was found between Mal and Con either for age (t_42_ = 0.73; p = 0.47), years of schooling (t_42_ = 1.38; p = 0.17), CPM score (t_42_ = 0.01; p = 0.99) and BNT score (t_42_ = -0.94; p = 0.35). All participants were literate. See Table 1 for participants' demographic information and questionnaires scores.

**Table 1:** Socio-demographic description of the samples.

		Mal	Con	Between-groups Differences
N. Tot		24	20	-
N. male		12	9	X_(1)_ = 0.11, p = .741
Age (years)		7.71 SE 0.32	7.35 SE 0.38	t_42_ = 0.73; p = .472
Age range (years)		5–10	5–10	-
Schooling (years)		2.50 SE 0.27	2 SE 0.23	t_42_ = 1.38; p = .174
BNT score		11.33 SE 0.98	12.60 SE 0.90	t_42_ = -0.94; p = .35
CPM score		18.66 SE 0.83	18.65 SE 0.82	t_42_ = 0.01; p = .99
First Language (%) *	Temne	54.17	5	X_(8)_ = 23.74, p = .003
	Mende	20.83	10	
	Limba	8.33	50	
	Krio	0	10	
	English	0	0	
	Other	16.67	25	
Homeless children (%)		100	0	-
Daytime spent on the street (hours) *		7.96 SE 0.55	2.8 SE 0.22	t_42_ = 7.84; p <.001
Night-time spent on the street (hours) *		8.38 SE 0.68	0.15 SE 0.11	t_42_ = 10.5; p <.001
Street-activities (%)	Provide food and shelter *	75	15	X_(1)_ = 15.7, p <.001
	Work *	75	0	X_(1)_ = 25.4, p <.001
	Robberies *	66.67	0	X_(1)_ = 20.9, p <.001
	Play	83.33	90	X_(1)_ = 0.41, p = .521
Health care coverage (%) ^a^ *		33.33	85	X_(1)_ = 11.8, p <.001
Access to basic needs (%) ^b^ *		25	90	X_(1)_ = 18.6, p <.001
Critical life events (%)	Physical Abuse *	62.5	10	X_(1)_ = 5.95, p = .015
	Sexual Abuse *	12.50	0	X_(1)_ = 2.68, p = .101
	Physical&Sexual Abuses *	12.50	0	X_(1)_ = 2.68, p = .101
	Mourning	75	50	X_(1)_ = 2.95, p = .086
Presence of an Adult Caregiver (%) *		8.33	100	X_(1)_ = 36.67, p <.001
Monthly family Income in Leone (%) *	<200,000 SLL	100	10	X_(3)_ = 33.4, p <.001
	200,000–500,000 SLL	0	25	
	500,000–700,000 SLL	0	35	
	>700,000 SLL	0	15	
Caregivers' Schooling (years)		-	4,6 SE 0.23	-
Caregivers' Employment (%)	Full-time salaried jobs ^c^	-	10	-
	Occasional job	-	20	-
	Trader	-	30	-
	Driver	-	5	-
	Artisan	-	15	-
	Miner	-	20	-

Maltreated children (Mal) and controls (Con) socio-demographic characteristics. Numbers may not add to total due to missing data or rounding. BNT: Boston Naming Test; CPM: Colored Progressive Matrices; SLL: Sierra Leonean Leone, currency of Sierra Leone.

* p < 0.05.

^a^–Health care coverage was defined as children’s access to preventive healthcare (i.e., vaccination, disease screening, malaria protection) and basic disease treatments (i.e., treatment of malaria, fever and diarrhea).

^b^–Access of basic needs was defined as children’s possibility to obtain adequate food, clean water, clothes and shelter.

^c^—Full-time salaried jobs include physician, nurse, educator, employee, social worker.

### Stimuli

Stimuli employed were 64 videos obtained by the Montreal Set of Facial Displays of Emotion [37] and already used in previous experiments conducted on African populations of different ages [7,8,38]. The stimuli were constructed by means of a face-morphing software (Squirlz Morph, http://www.xiberpix.net/SqirlzMorph.html), using one neutral facial expression as start image, and one emotional facial expression of the same actor, as end image. Each video, lasting 3 sec (15 fps; 800×560 pixels), showed the transition from a neutral facial expression to an emotional one (16 anger, 16 fear, 16 joy and 16 sadness). Each emotion expression was modeled by Asian, African, Hispanic and Caucasian actors balanced for gender (i.e., 4 stimuli for each ethnic group, 2 males and 2 females). For an exemplificative stimulus employed in the present study see S1 Stimulus. E-Prime 2.0 software (Psychology Software Tools, Inc.) was used for stimuli presentation.

### Procedure

The experimental protocol was approved by the Ethic Committee of the Ministry of Health and Sanitation of the Republic of Sierra Leone and it was in line with the Declaration of Helsinki 2013. All reported data were collected from October to December 2013. To avoid confounding effects, participants were all recorded in the morning, two hours after food intake. All participants were tested in the same location and using the same experimental setting. A local social-worker was always present to ensure that participants remained at ease, understood the instructions and to translate from English to Krio, if necessary. Children sat comfortably at a table in front of a monitor (1024X768@75Hz), they were asked to carefully observe the video stimuli. The experiment consisted of four ‘‘condition-blocks” (each lasting 192 sec) and two ‘‘baseline blocks” (each lasting 120 sec). The four condition-blocks, one for each emotional condition (i.e., anger, fear, joy and sadness), were randomly presented. Inside each condition-block the sixteen stimuli, comprising the same emotion, were randomly presented three times (i.e., 48 trials at each block). Each stimulus was preceded by a fixation cross lasting 1 second. The two baseline-blocks, consisting of a black centered fixation cross on gray background, were performed one before (baseline) and one after (recovery) the four condition-blocks. Overall the experiment lasted 17 min. In order to maintain participants’ attention, after each condition-block the experimenter posed a question about the videos just shown. Participants’ faces were video-recorded to ensure that they looked at the screen and to facilitate the subsequent artifacts detection.

Facial EMG and RSA were recorded for the entire duration of the experiment. Data were converted and amplified with an eight-channel amplifier (PowerLab8/30; ADInstruments UK) and displayed, stored, and reduced with LabChart 7.3.1 software package (ADInstruments, 2011).

*Facial EMG—*4 mm Ag/Ag-Cl electrodes were bipolarly attached on the left side of the face over corrugator supercilii and zygomaticus major muscle regions [39]. Participants’ skin was cleaned and prepared by alcohol solution and the electrodes were filled with gel conductive paste. Facial EMG was sampled at 2 kHz and recorded with an online Mains Filter (adaptive 50 Hz filter). A 20–500 Hz band-pass filter [40] was applied offline on the raw facial EMG signal. Following standard practice, the average amplitude of the EMG signal was obtained via root-mean-square method and the EMG response (expressed in microvolts, μV) was computed as change scores between the activity during each 500 msec of the 3 sec stimulus period and the 500 msec of the fixation cross immediately preceding stimulus onset [39]. EMG signal was screened for artifacts by a blind coder who firstly deleted trials with artifacts due to electrical noise (less than 3.5% of removed trials), and subsequently, inspected participants' face videos to remove trials affected by motion artifacts (i.e., a variety of facial movements not directly related to stimuli observation but affecting the EMG signal like cough, sneeze, yawn) and trials in which participants did not directly observe the screen. Overall, form the initial sample (64 participants) 18 children (10 maltreated children and 8 controls) were excluded due to more than 30% of trials removed. Considering the final sample (44 participants), the total average percentage of removed trials was 17,96% SE 1.08, without significant differences between the two groups (Mal: 16.67% SE 1.67; Con 19.5% SE 1.29; F_(1,42)_ = 0.549, p = 0.463) and between conditions (Anger: 19.19% SE 0.694; Fear: 18.74, SE 0.662; Joy: 19.32, SE 0.734; Sad: 18.03; SE 0.599; F_(3,42)_ = 0.224, p = 0.880).

*RSA—*Three 10 mm Ag/AgCl pre-gelled electrodes (ADInstruments, UK) were placed in an Einthoven's triangle configuration. The ECG was sampled at 1 kHz and online filtered with the Mains Filter. The peak of the R-wave of the ECG was detected from each sequential heartbeat. The R-R intervals were extracted and the artifacts edited by integer division or summation [29]. Editing consisted of visual detection of outlier points, typically caused by failure to detect an R-peak (e.g., edit via division) or faulty detections of two or more peaks within a period representing the R-R interval (e.g., edit via summation). The amplitude of RSA was quantified with CMetX (available from http://apsychoserver.psych.arizona.edu) that produces data with a correlation near the unity with those obtained using Boher & Porges method [41]. The amplitude of RSA [expressed in ln(msec)2] was calculated as the variance of heart rate activity across the band of frequencies associated with spontaneous respiration in children (0.24–1.04 Hz). RSA was extracted for the entire duration of each condition-block and each baseline-block, according to guidelines [29]. To assure an homogeneous computation of RSA amplitude standard procedure was conducted on consecutive epochs lasting 30 sec both for baseline, recovery and for each condition-block. Hence, the baseline and recovery RSA values resulted from the average of 4 consecutive epochs, whereas the condition-blocks RSA values was obtained by the mean of 6 consecutive epochs. The RSA suppression value for each condition-block was measured as a change of scores between the RSA of the condition-block and baseline RSA value.

### Statistical data analyses

To verify between-groups differences in facial EMG activations during the visualization of facial expressions of emotions, two separate repeated-measures ANOVAs, one for each recorded muscle (corrugator and zygomaticus), were conducted with Group (Mal, Con) and Sex (M, F) as between-factors and with Emotion (anger, fear, joy, sadness) and Epoch (6 epochs, each lasting 500msec) as within factors.

As mention in the introduction, a robust literature describe facial mimicry phenomenon as a congruent muscular response [see for example, 12,16,32–34] to others’ facial expressions of both positive and negative emotions. Thus, corrugator EMG responses are especially expected for facial expressions of negative emotions (e.g., anger, fear and sadness), whereas, zygomaticus EMG responses are attended for facial expressions of positive emotions (e.g., joy). This congruent muscular activation is confirmed also by the present results where differentiate muscular responses were found both for corrugator and zygomaticus muscles in response to negative and positive emotions, respectively (see the significant main effect of Emotion factor showed in the repeated-measures ANOVAs conducted on corrugator and zygomaticus muscles). In order to better investigate between-groups differences in congruent facial mimicry responses, further repeated-measures ANOVAs were conducted on corrugator EMG activations in response only to facial expressions of negative emotions and, conversely, on the zygomaticus EMG activations in response to facial expressions of positive emotion. Specifically, two repeated-measures ANOVAs were performed, the first one on corrugator EMG activations in response only to negative facial expressions, with Group (Mal, Con) and Sex (M, F) as between-factors, and with Negative Emotion (anger, fear, sadness) and Epoch (6 epochs, each lasting 500msec) as within factors. The second one on zygomaticus EMG activations in response to joy facial expressions, with Group (Mal, Con) and Sex (M, F) as between-factors, and with Epoch (6 epochs, each lasting 500msec) as within factors.

Accordingly to guidelines [42], when the sphericity assumption was violated, Geisser–Greenhouse correction was calculated and adjusted df, corrected p values, and epsilon values (Ɛ) reported. Whenever appropriate, significant between- and within-group differences were explored performing Tukey post-hoc comparisons. Partial eta square (ƞ^2^_p_) was calculated as effect size measure.

To investigate possible between-groups differences in RSA values recorded before and after the experiment execution, two independent sample t-tests (two-tailed), one for baseline RSA values and one for recovery RSA values were performed comparing the two experimental groups. Since, no significant differences between groups were found, to investigate possible experiment influence on RSA values a dependent sample t-test (two tailed) was conducted contrasting baseline and recovery RSA values of all participants, regardless of group membership. Bonferroni correction for multiple comparisons was applied on p_s_ significant levels.

Finally, a detailed analysis of RSA suppression values was performed. In order to verify the existence of a significant correlation between baseline RSA and the RSA suppression values measured in response to the different conditions, two Pearson’s correlations analyses (one for each group) were performed. For each correlation analysis baseline values and the RSA suppression values of each condition were correlated [43]. Bonferroni correction for multiple comparisons was applied on p_s_ significant levels.

S1 Dataset reports the dependent variables included in the described statistical data analyses.

## Results

### Facial EMG

#### Corrugator

Mauchly’s test conducted on corrugator EMG activity showed a violation of sphericity assumption for Epoch factor (χ^2^_(14)_ = 92.92, p< 0.001). Hence, degrees of freedom were adjusted using Greenhouse-Geisser correction (Ɛ = 0.485). Repeated-measures ANOVA conducted on corrugator EMG activity revealed a significant main effect of the factor Emotion (F_3,120_ = 5.12, p = 0.002; ƞ^2^_p_ = 0.11) and a significant interaction Emotion by Group (F_3,120_ = 3.46, p = 0.02; ƞ^2^_p_ = 0.08). No significant main effect was estimated for Group (F_1,40_ = 1.13, p = 0.294; ƞ^2^_p_ = 0.03), Sex (F_1,40_ = 0.001, p = 0.998; ƞ^2^_p_ = 0.00) and Epoch (F_2.425,97.008_ = 2.48, p = 0.078; ƞ^2^_p_ = 0.06).

**Fig 1:**
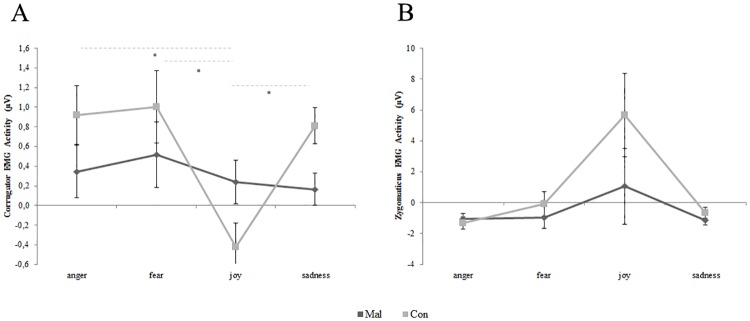
Corrugator and zygomaticus EMG responses to facial expressions of positive and negative emotions. **A)** Corrugator EMG activity displayed emotion by emotion for maltreated group (Mal) and control group (Con). * = p < 0.05. Error bars represent SE. **B)** Zygomaticus EMG activity displayed emotion by emotion for maltreated group (Mal) and control group (Con).

Post-hoc comparisons conducted on the main effect of Emotion revealed that corrugator EMG activity recorded in response to joy facial expressions (-0.09 μV, SE 0.16) was significantly lower than corrugator EMG activity measured in response to both angry (0.63 μV, SE 0.20; p = 0.03), and fear facial expressions (0.75 μV, SE 0.25; p = 0.01). No significant difference was found between joy and sadness EMG activity (0.48 μV, SE 0.12; p = 0.14). Post-hoc comparisons performed on Emotion by Group interaction (Fig 1A) showed that only among controls the corrugator EMG activity in response to joy facial expression was significantly lower than that recorded in response to all other negative facial expressions (all p_s_ < 0.05). No significant differences were found among maltreated children in corrugator EMG activity during positive and negative facial expressions visualization (all p_s_ > 0.05).

**Fig 2:**
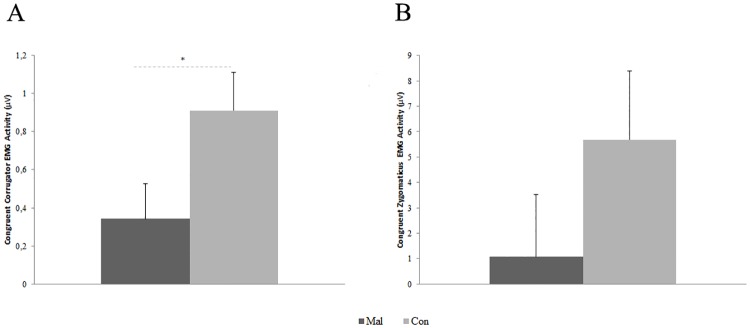
Corrugator and zygomaticus congruent EMG responses to facial expressions of emotions. **A)** Mean congruent corrugator EMG activity for maltreated group (Mal) and control group (Con) during the visualization of facial expressions of negative emotions. * = p < 0.05. Error bars represent SE. **B)** Mean congruent zygomaticus EMG activity for maltreated group (Mal) and control group (Con) during the visualization of joy facial expressions.

Mauchly’s test conducted on corrugator EMG activity considering only negative emotions showed a violation of sphericity assumption for Epoch factor (χ^2^_(14)_ = 95.41, p< 0.001). Hence, degrees of freedom were adjusted using Greenhouse-Geisser correction (Ɛ = 0.489). Repeated-measures ANOVA conducted on corrugator EMG activity in response only to facial expressions of negative emotions revealed a significant main effect of the factor Group (F_1,40_ = 4.33, p = 0.044; ƞ^2^_p_ = 0.01) (Fig 2A). No significant main effect was estimated for Sex (F_1,40_ = 0.10, p = 0.754; ƞ^2^_p_ = 0.002), Emotion (F_2,80_ = 0.61, p = 0.547; ƞ^2^_p_ = 0.01) and Epoch (F_2.425,97.008_ = 1.67, p = 0.188; ƞ^2^_p_ = 0.04). Post-hoc comparison performed on the main effect of Group showed a significantly higher corrugator EMG activity among controls with respect to maltreated children, independently from the facial expressions of negative emotions displayed (p = 0.04).

#### Zygomaticus

Mauchly’s test conducted on zygomaticus EMG activity showed a violation of sphericity assumption for Emotion (χ^2^_(5)_ = 153.27, p< 0.001) and Epoch factors (χ^2^_(14)_ = 326.51, p< 0.001). Hence, degrees of freedom were adjusted using Greenhouse-Geisser correction (Emotion: Ɛ = 0.37; Epoch: Ɛ = 0.23; Emotion by Epoch: Ɛ = 0.09). Repeated measures ANOVA conducted on zygomaticus EMG activity revealed only a significant effect of the factor Emotion (F_1.12,44.98_ = 5.79, p = 0.017; ƞ^2^_p_ = 0.13). No significant main effect was estimated for Group (F_1,40_ = 1.51, p = 0.226; ƞ^2^_p_ = 0.04), Sex (F_1,40_ = 1.82, p = 0.185; ƞ^2^_p_ = 0.04) and Epoch (F_1.152,46.093_ = 2.30, p = 0.132; ƞ^2^_p_ = 0.05). Furthermore, also the interaction Emotion by Group (F_3,120_ = 1.49, p = 0.221; ƞ^2^_p_ = 0.04) was not significant (Fig 1B). Post-hoc comparisons conducted on the significant main effect of Emotion revealed, as expected, that zygomaticus EMG activity recorded in response to joyful facial expressions (3.37 μV, SE 1.82) was significantly higher than zygomaticus EMG activity measured in response to both anger (-1.19 μV, SE 0.27; p = 0.01), fearfulness (-0.51 μV, SE 0.53; p = 0.03) and sadness facial expressions (-0.89 μV, SE 0.23; p = 0.01).

Mauchly’s test conducted on zygomaticus EMG activity considering only positive emotion showed a violation of sphericity assumption for Epoch factor (χ^2^_(14)_ = 450.48, p< 0.001). Hence, degrees of freedom were adjusted using Greenhouse-Geisser correction (Ɛ = 0.213). Repeated-measures ANOVA conducted on zygomaticus EMG activity in response only to facial expressions of joy demonstrated the absence of significant main effects of the factor Group (F_1,40_ = 1.60, p = 0.213; ƞ^2^_p_ = 0.04) (Fig 2B), Sex (F_1,40_ = 1.65, p = 0.206; ƞ^2^_p_ = 0.04), and Epoch (F_1.066,42.627_ = 2.87, p = 0.095; ƞ^2^_p_ = 0.07).

### RSA

#### Baseline and recovery RSA

Bonferroni corrected t-tests (with α 0.05 = 0.016) comparing Mal and Con participants’ RSA values at baseline [STch: 5.90 ln(msec)^2^, SE 0.34; Con: 6.15 ln(msec)^2^, SE 0.27] was not significant (t_42_ = -0.55, p = 0.58). Similarly, Bonferroni corrected t-tests contrasting Mal and Con participants’ RSA values at recovery [STch: 5.90 ln(msec)^2^, SE 0.28; Con: 6.08 ln(msec)^2^, SE 0.26] were not significant (t_39_ = -0.46, p = 0.65).

Considering all participants regardless of group membership, Bonferroni corrected t-test comparing baseline [6.01 ln(msec)^2^, SE 0.22) and recovery RSA (5.98 ln(msec)^2^, SE 0.19] values were not significant (t_83_ = -0.11, p = 0.91).

#### RSA suppression

**Fig 3:**
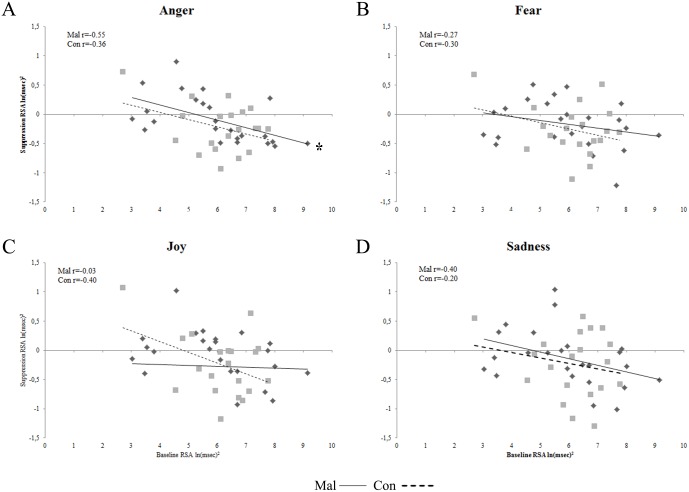
Correlation plots between baseline and suppression RSA values in the two groups. Plots of correlations between baseline and suppression RSA values for maltreated children (Mal) and controls (Con) displayed emotion by emotion. * = Bonferroni corrected p < 0.012.

Bonferroni corrected (with α 0.05 = 0.012) two-tailed Pearson’s correlations performed for Mal group demonstrated a significant negative correlation between baseline RSA and suppression RSA values in response to angry facial expressions (r_24_ = -0.55; p = 0.005) (Fig 3A). On the contrary, Bonferroni corrected (with α 0.05 = 0.012) two-tailed Pearson’s correlations performed on Con group revealed an absence of any significant relation between baseline RSA and suppression RSA values in response to all facial expressions of emotions (all p_s_ > 0.08) (Fig 3). For a description of the suppression RSA values obtained by the two groups in response to the different facial expressions of emotions, please see Table 2.

**Table 2:** Suppression RSA values in response to facial expressions of emotions of Mal and Con groups.

		Suppression RSA values			
Group		*Mean*	*SE*	*95% C*.*I*.	
Mal	anger	-,085	,084	-,25	,09
	fear	-,165	,082	-,33	,00
	joy	-,271	,170	-,61	,07
	sadness	-,133	,100	-,34	,07
Con	anger	-,222	,093	-,41	-,03
	fear	-,247	,090	-,43	-,06
	joy	-,220	,187	-,60	,16
	sadness	-,210	,110	-,43	,01

Suppression RSA values (mean, SE, 95% C.I.) for maltreated children (Mal) and controls (Con) showed in response to the different facial expressions of emotions. SE = Standard Error; 95% C.I. = 95% Confidence Interval.

## Discussion

The aim of the present study was to investigate facial mimicry responses and vagal regulation to the facial expressions of positive and negative emotions among maltreated children. To this aim spontaneous facial EMG activities and RSA values were recorded during the visualization of the facial expressions of positive and negative emotions.

Results demonstrate that maltreated children show specific alterations of the corrugator spontaneous mimicry to facial expressions of emotions. First, considering the corrugator mimicry to the facial expressions of both positive and negative emotions, maltreated children did not manifest the typical positive-negative modulation, shown instead by control participants. As mention in the introduction, this differentiated response occurs spontaneously [17] and it is related to valence attribution to others’ facial expressions of emotions [18,19].

Second, when only negative facial expressions were taken into account, maltreated children demonstrated lower corrugator mimicry responses than control participants.

Suppressed congruent corrugator mimicry was already found among adolescents exposed to high levels of maltreatment [7] and among women victims of childhood sexual abuse [31]. These results illustrate the consistent influence of childhood maltreatment and childhood trauma on the spontaneous and congruent facial mimicry responses to others’ facial expressions of negative emotions. Conversely, one previous study showed an increment of congruent corrugator mimicry among abused children [30]. This opposite result could be explained by differences in the employed paradigm. First, in this study participants took part to a direct hostile interplay with a peer whereas, both in the present and in previous studies showing a suppression of congruent corrugator mimicry, participants were involved in a passive observation task. Furthermore, and not less importantly, the age of people expressing emotions was different between the study showing a higher corrugator response and the studies demonstrating reduced corrugator mimicry. This factor represents a crucial aspect when young people’s facial mimicry responses to others’ facial expressions are considered [44]. Bearing in mind these aspects, all these results might suggest a different impact of maltreatment on victims’ spontaneous facial mimicry depending on the nature of the relation in which they are involved and on the age of their interlocutors. It is also important to note that, in the present study, zygomaticus facial mimicry was not significantly influenced by maltreatment exposure. In fact, both groups showed the expected positive-negative modulation in zygomaticus spontaneous mimicry to facial expressions of positive and negative emotions. Furthermore, considering only the congruent facial mimicry of the zygomaticus muscle, even if maltreated children showed a lower amplitude of zygomaticus responses to the facial expression of positive emotion than controls, this difference was not significant. Joy is the best recognized facial expression among both maltreated and non-maltreated children [7,9,44], moreover it is the first basic emotion to be recognized [45] and the first one to be spontaneously imitated [46]. The preservation of zygomaticus facial mimicry thus demonstrates that facial mimicry response to positive facial expressions is more resistant to external environmental influences than corrugator facial mimicry to negative ones.

With respect to vagal regulation, although the two groups did not vary for their RSA values at rest, maltreated and control children showed different relations between RSA at rest and RSA suppression values in response to facial expression of anger. On the basis of previous studies [7,32] and considering the vagal functional regulation in social context [27,47], people with higher baseline RSA should show greater RSA suppression to meet metabolic demands of taxing and hostile environmental cues, like angry facial expressions. On the contrary, no significant relation between baseline RSA and RSA suppression values is anticipated in response to the facial expression of joy, fear and sadness. Indeed, joy is a socially engaging expression whereas fear and sadness are facial expressions of negative, but not directly adverse, emotions. Unexpectedly, whereas control children did not show any significant relation between baseline RSA and RSA suppression values in response to facial expressions of anger, maltreated children manifested the functional and expected inverse correlation between baseline RSA and RSA suppression values recorded during the visualization of angry facial expressions. These results suggest that the hostile and adverse life conditions to which maltreated children have to confront, foster an earlier functional adaptation of autonomic regulation to a challenging social context. In other words, the exposure to negative and aggressive environments might induce an earlier development of the functional synchronization between vagal regulation and the external threatening environment, which, in normal conditions is not established.

In conclusion, the present study demonstrates a different impact of childhood maltreatment on facial mimicry and vagal regulation during the perception of facial expressions of emotions. Maltreatment induces the alteration and suppression of the spontaneous facial mimicry to the facial expressions of emotions, fostering a functional reduction of one of the physiological mechanisms contributing to the empathic understanding of others’ emotions. At the same time, childhood maltreatment forces a prompt development of the functional synchronization between vagal regulation and threatening stimuli coming from the external environment, hence promoting the rapid recruitment of fight-or-flight defensive behavioral strategies. The present data seem to draw a scenario where an early functional adaptation to the hostile environment of the two investigated physiological mechanisms leads to divergent development of victims’ emphatic and self-regulation social skills. The clinical observations concerning victims’ conduct disorders and aggressive behaviors should find new frames of reference to plan new therapeutic and rehabilitative programs directed to children victims of maltreatment.

Some limitations of the study have to be described. First, even though the sample size assures the power of statistics adopted, it was less than 50 participants. Second, the maltreated group was composed by children suffering from different forms of maltreatment and, probably, with different degrees of impact. Due to the small sample size and to difficulties in recruiting consistent information, the rigorous investigation of the specific effect of type and number of abuses is not possible. Finally, the lack of validated and applicable scales on underage African samples prevented, in the present study, the formal assessment and correlation of the potential psychiatric sequelae generally following maltreatment exposure (i.e., PTSD, conduct disorder and depression). Further studies might address this point trying to directly relate physiological and behavioral deficits in maltreated populations of disadvantaged African children.

## Supporting Information

S1 Dataset Dataset of participants’ EMG activities and RSA responses to facial expressions of emotions. (XLSX)

S1 Stimulus Exemplificative stimulus employed in the present study derived from the Montreal Set of Facial Displays of Emotion. (AVI)
